# Protective effect of *Macleaya cordata* isoquinoline alkaloids on lipopolysaccharide-induced liver injury in broilers

**DOI:** 10.5713/ab.23.0267

**Published:** 2023-11-01

**Authors:** Jiaxin Chen, Weiren Yang, Hua Liu, Jiaxing Niu, Yang Liu, Qun Cheng

**Affiliations:** 1 Department of Animal Science, Qingdao Agricultural University, Qingdao 266109, China; 2 Key Laboratory of Efficient Utilization of Non-grain Feed Resources (Co-construction by Ministry and Province), Ministry of Agriculture and Rural Affairs, Shandong Provincial Key Laboratory of Animal Biotechnology and Disease Control and Prevention, Department of Animal Science and Veterinary Medicine, Shandong Agricultural University, Tai’an 271018, China; 3 College of Animal Science and Technology, Hunan Agriculture University, Changsha 410128, China

**Keywords:** Broiler, Isoquinoline Alkaloids, Lipopolysaccharide, Liver Injury, *Macleaya Cordata*

## Abstract

**Objective:**

This experiment aimed to explore the protective action of dietary supplementation with isoquinoline alkaloids (IA) from *Macleaya cordata* on lipopolysaccharide (LPS)-induced liver injury in broilers.

**Methods:**

Total 216 healthy broilers were selected in a 21-d trial and assigned randomly to the following 3 treatments: control (CON) group, LPS group, and LPS+IA group. The CON and LPS groups were provided with a basal diet, whereas the LPS+IA group received the basal diet supplemented with 0.6 mg/kg *Macleaya cordata* IA. Broilers in LPS and LPS+IA groups were intraperitoneally injected with LPS (1 mg/kg body weight) at 17, 19, and 21 days of age, while those in CON group were injected with equivalent amount of saline solution.

**Results:**

Results showed LPS injection caused systemic and liver inflammation in broilers, inhibited immune function, and ultimately lead to liver injury. By contrast, supplementation of IA ameliorated LPS-induced adverse change in serum parameters, boosted immunity in LPS+IA group. Furthermore, IA suppressed the elevation of hepatic inflammatory cytokines and caspases levels induced by LPS, as well as the expressions of genes related to the toll-like receptor 4 (TLR4)/myeloid differentiation primary response 88 (MyD88)/nuclear factor-kappa B (NF-κB) pathway.

**Conclusion:**

Dietary inclusion of 0.6 mg/kg *Macleaya cordata* IA could enhance immune function of body and inhibit liver damage via inactivating TLR4/MyD88/NF-κB signaling pathway in broilers.

## INTRODUCTION

The liver plays a key role in the metabolism of nutrients, regulation of immune system, decomposition of chemicals and drugs, and other physiological functions [1]. At present, the intensification and scale of poultry breeding improves the efficiency and profit of poultry production, but the risk of liver damage to broilers also increases due to the effects of environment, pathogenic bacteria and feed mycotoxin contamination [2]. Especially, bacterial infection-induced liver damage is very common in modern broiler chicken production [3,4]. In the past, antibiotic therapy was an effective method of liver protection [5]. However, antibiotics have been banned for use in animal production and restricted to the therapeutic use in view of the livestock health and food safety caused by the abuse of antibiotic feed additives [6]. Therefore, looking for alternatives to antibiotics has become a hot spot in animal production in recent years [2,7].

*Macleaya cordata* is a perennial herb medicinal plant. Numerous studies have demonstrated that its extracts possess a variety of biological activities, including anti-inflammatory, antioxidant, and immune regulation, and remarkable efficacy in enhancing the production of all kinds of livestock and poultry [7,8]. Benzophenanthridine alkaloids (BA; sanguinarine and chelerythrine) and isoquinoline alkaloids (IA; protopine and allocryptopine) are the main primary constituents found in extracts of *Macleaya cordata* [9]. Currently, there has been extensive research on the impact of *Macleaya cordata* BA on animal production and health status [9–11], however, there is a limited amount of evaluation of *Macleaya cordata* IA in animal production. The IA from *Macleaya cordata* have recently been approved as a veterinary drug, named as Bopu Powder (veterinary drug No.180415374), for the treatment of *Escherichia coli* (*E. coli*)-induced chicken diarrhea [12]. A previous study in laying hens showed that 1.5 to 6 mg/kg IA addition had no additive effects, while dietary 0.38 to 0.75 mg/kg IA supplementation had a greater impact on egg quality and antioxidant status [12]. Liu et al [7] indicated that including 0.6 mg/kg of IA derived from *Macleaya cordata* in the broiler diet could significantly enhance growth performance and liver health of broilers. Moreover, the intestinal development and physiological function of broilers were improved by *Macleaya cordata* IA addition to the basal diet, characterized by reduction of inflammatory response, improvement of antioxidant capacity, and increase of beneficial bacteria abundances in the intestine [13]. Nevertheless, there is little relevant literature on whether dietary supplementation of *Macleaya cordata* IA can relieve liver injury of broilers.

As the composition of Gram-negative bacteria membrane structure, lipopolysaccharide (LPS) can stimulate monocyte macrophages to secrete inflammatory cytokines causing inflammatory damage to the liver [14,15]. Huang et al [16] has proved that intraperitoneal administration of 50 mg/kg body weight (BW) LPS enhanced inflammatory cell infiltration and cell apoptosis of broiler liver tissue by activating toll-like receptor 4 (TLR4) signaling pathway, leading to acute injury of broiler liver. Moreover, recent study in broilers indicated that dietary addition with IA from *Macleaya cordata* could decreased hepatic inflammation via inhibition of TLR4/myeloid differentiation primary response 88 (MyD88)/nuclear factor-kappa B (NF-κB) signaling pathway [7]. Therefore, this study aimed to explore the potential of dietary *Macleaya cordata* IA supplementation to alleviate liver injury after an LPS challenge in broilers based on TLR4/MyD88/NF-κB signaling pathway.

## MATERIALS AND METHODS

The animal study protocol was approved by the Ethics Committee of Qingdao Agricultural University (protocol code QAU20220103195).

### Animal and treatments

A total of 216 Arbor Acres (AA) broilers, healthy and 1-day-old with similar BW (48.35±0.41 g), were divided randomly into three treatments devoted control (CON) group, LPS group, and LPS+IA group, respectively. Each treatment group had 6 replicates with 12 broilers in each replicate. Broilers in the CON group and LPS group were provided with a basal diet, whereas broilers in the LPS+IA group were given a basal diet added with 0.6 mg/kg IA which was extracted from *Macleaya cordata* [7]. The IA is provided by the Hunan Meikeda Biological Resources (Changsha, China), and the ratio of protopine to allotypotopine is 2:1 [12,13]. The basal diet (Table 1) was prepared following the broiler nutritional requirements recommended by the Chinese Ministry of Agriculture (2004). The trial lasted for 21 days. At 07:00 am on days 17, 19, and 21, the broilers of LPS group and LPS+IA group were injected with 0.5 mL of LPS (1 mg/kg BW; L2880, *E. coli* O55:B5; Sigma-Aldrich, St. Louis, MO, USA), while the chickens of the CON group were injected with 0.9% sterile saline of the equivalent volume [17,18]. During the whole experiment, all broilers were kept in a three-level cages in a light- and temperature-controlled room, and had free access to feed and water.

### Sample collection

On day 21 of the trial, one broiler per replicate was selected, with BW closest to the average weight of each replicate, and blood samples (10 mL) were collected from wing vein after 3 hours of LPS challenge. These samples were collected into vacuum tubes without heparin sodium, and the supernatant was divided into 1.5 mL EP tubes after centrifugation at 3,500 g and stored in −35°C until further analysis. Afterwards, the broilers were euthanized and about 2 g liver samples were collected. Part of the liver samples was cut up and loaded into 2 mL frozen storage tubes, and then stored at −80°C after being frozen quickly with liquid nitrogen; the other part was fixed with 4% paraformaldehyde solution at room temperature for 24 hours.

### Liver histopathological examination

Liver tissue samples were dehydrated using various concentrations of ethyl ethanol and then embedded in paraffin wax after being fixed with a 4% paraformaldehyde solution for 24 hours. Afterward, the liver tissues embedded in paraffin were sliced into sections measuring 5 micrometers and then subjected to staining with hematoxylin and eosin (H&E) to facilitate morphological analysis. In the end, liver sections were observed through the use of a digital microscope (Olympus BX 51, Tokyo, Japan).

### Determination of serum biochemical parameters concentrations

The levels of total protein (TP), albumin (ALB), high density lipoprotein (HDL), total cholesterol (TCHO), low density lipoprotein (LDL), triglycerides (TG), glucose (GLU), urea nitrogen (UREA), and alanine aminotransferase (ALT) in the serum were examined using commercial kits (Jiancheng Bioengineering Institute, Nanjing, China) on a Roche automated biochemical analyzer (Roche Diagnostic System Inc., Indianapolis, IN, USA).

### Determination of serum immunoglobulins and complements concentrations

Commercial enzyme-linked immunosorbent assay (ELISA) kits (Meimian Industrial Co., Ltd, Jiangsu, China) were used to determine serum levels of immunoglobulin A (IgA), immunoglobulin G (IgG), immunoglobulin M (IgM), and complements C3 and C4. The measurement procedures were performed according to the description in Chen et al [19].

### Determination of inflammatory cytokines concentrations and hepatic caspases activities

The levels of interleukin (IL)-1β, tumor necrosis factor α (TNF-α), IL-4, IL-6, and IL-10 in the serum, along with the concentrations of NOD-like receptor family pyrin domain containing 3 (NLRP3), TNF-α, IL-1β, IL-6, and IL-18 in the liver were determined using ELISA kits purchased from R&D Systems Inc. (Minneapolis, MN, USA), and all test steps were performed according to Chen et al [20]. The levels of caspase-3 and caspase-1 was measured with the ELISA kits purchased form Jiancheng Bioengineering Institute following the protocol described in Li et al [14].

### Determination of gene expression in liver

The extraction of total RNA from the liver samples used the AG RNAex Pro reagent (Accurate Biology, Hunan, China). The cDNA was synthesized by the reverse transcription (RT) kit (Accurate Biology, China), and real-time quantitative polymerase chain reaction (RT-qPCR) was amplified with SYBR Green Premix Pro Taq HS qPCR Kit (Accurate Biology, China). The mRNA expression levels of all primers, including TLR4, MyD88, NF-κB, B-cell-lymphoma-2 (Bcl-2), and Bcl-2-associated X (Bax) were examined using a LightCycler 96 (Roche Basel, Switzerland). All primers sequences are listed in Table 2. The expression of the actin-associated gene target mRNA was calculated using the β-actin as an internal reference gene with 2^−ΔΔCT^ method [21].

### Statistical analysis

To test the statistical variances among treatments, a one-way analysis of variance was conducted using SAS 9.4 (Institute Inc., Cary, NC, USA) after assessing the data’s normal distribution using Shapiro-Wilk statistics (W>0.05). And the least significant procedure was used for multiple comparison analysis. All data were expressed as the mean±standard error in the figures. Significant differences are identified using * p< 0.05, ** p<0.01, and *** p<0.001, and ^#^ 0.05<p<0.10 is considered as a significant trend. The “ns” is considered as non-significant differences.

## RESULTS

### Serum concentrations of biochemical parameters

**Figure 1:**
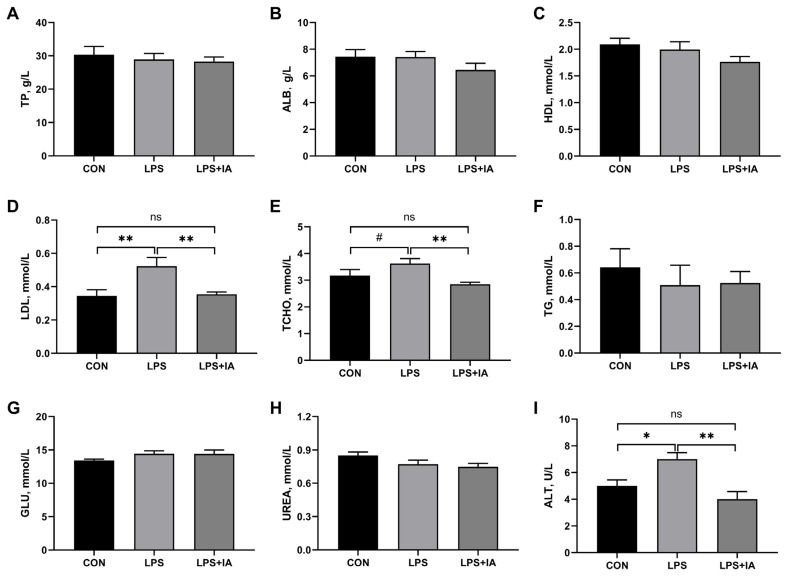
Effects of dietary *Macleaya cordata* isoquinoline alkaloids (IA) on serum concentrations of biochemical parameters in broilers challenged with lipopolysaccharide (LPS). (A) Total protein (TP); (B) Albumin (ALB); (C) High density lipoprotein (HDL); (D) Low density lipoprotein (LDL); (E) Total cholesterol (TCHO); (F) Triglycerides (TG); (G) Glucose (GLU); (H) Urea nitrogen (UREA); (I) Alanine transaminase (ALT). CON, broilers given a basal diet; LPS, LPS-challenged broilers given a basal diet; LPS+IA, LPS-challenged broilers given a basal diet supplemented with 0.6 mg/kg IA extracted from *Macleaya cordata*. All data were expressed as the mean±standard error in the figures. Significant differences were indicated using * p<0.05 and ** p<0.01, and ^#^ 0.05<p<0.10 is considered as a significant trend. The “ns” is considered as non-significant differences.

As shown in Figure 1, relative to the CON group, LPS administration significantly increased serum concentrations of LDL (Figure 1D) and ALT (Figure 1I) (p<0.05), and tended to increase serum TCHO (Figure 1E) concentration of broilers (p<0.10). Supplementing the diet with IA inhibited LPS-induced increases in serum concentrations of LDL, TCHO, and ALT to the levels observed in the CON group (p>0.05). The three groups did not show any notable variations in the serum levels of TP (Figure 1A), ALB (Figure 1B), HDL (Figure 1C), TG (Figure 1F), GLU (Figure 1G), and UREA (Figure 1H) (p>0.05).

### Serum inflammatory cytokines concentrations

**Figure 2:**
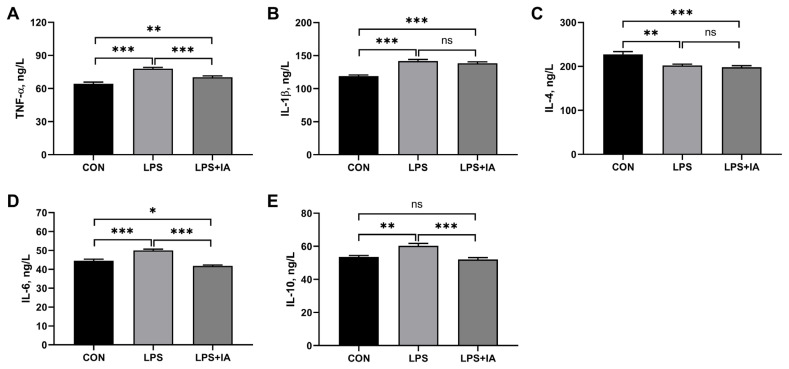
Effects of dietary *Macleaya cordata* isoquinoline alkaloids (IA) on serum inflammatory cytokines concentrations in broilers challenged with lipopolysaccharide (LPS). (A) Tumor necrosis factor α (TNF-α); (B) Interleukin-1β (IL-1β); (C) Interleukin-4 (IL-4); (D) Interleukin-6 (IL-6); (E) Interleukin-10 (IL-10). CON, broilers given a basal diet; LPS, LPS-challenged broilers given a basal diet; LPS+IA, LPS-challenged broilers given a basal diet supplemented with 0.6 mg/kg IA extracted from *Macleaya cordata*. All data were expressed as the mean±standard error in the figures. Significant differences were indicated using * p<0.05, ** p<0.01, and *** p<0.001, and ^#^ 0.05<p<0.10 is considered as a significant trend. The “ns” is considered as non-significant differences.

As displayed in Figure 2, compared with the CON group, broiler chickens in the LPS group showed significantly higher serum TNF-α (Figure 1A), IL-1β (Figure 1B), IL-6 (Figure 1D), and IL-10 (Figure 1E) concentrations (p<0.05), but significantly lower serum IL-4 (Figure 1C) level (p<0.05). In LPS-challenged broilers, IA supplementation significantly decreased serum concentrations of TNF-α, IL-6, and IL-10 (p<0.05). Meanwhile, LPS+IA group showed significantly higher serum TNF-α and IL-1β concentrations (p<0.05) and significantly lower serum IL-4 and IL-6 concentrations (p< 0.05) than CON group, while there was no significant difference in serum IL-10 concentration between the CON and LPS+IA groups (p>0.05).

### Serum immunoglobulins and complements levels

**Figure 3:**
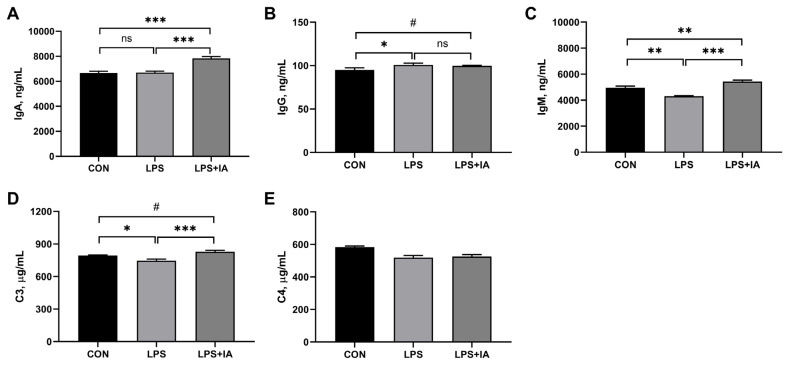
Effects of dietary *Macleaya cordata* isoquinoline alkaloids (IA) on serum immunoglobulins and complements levels in broilers challenged with lipopolysaccharide (LPS). (A) Immunoglobulin A (IgA); (B) Immunoglobulin G (IgG); (C) Immunoglobulin M (IgM); (D) Complement C3; (E) Complement C4. CON, broilers given a basal diet; LPS, LPS-challenged broilers given a basal diet; LPS+IA, LPS-challenged broilers given a basal diet supplemented with 0.6 mg/kg IA extracted from *Macleaya cordata*. All data were expressed as the mean±standard error in the figures. Significant differences were indicated using * p<0.05, ** p<0.01, and *** p<0.001, and ^#^ 0.05<p<0.10 is considered as a significant trend. The “ns” is considered as non-significant differences.

As displayed in Figure 3, significantly higher serum IgG (Figure 1B) concentration (p<0.05) and significantly lower serum IgM (Figure 1C) and complement C3 (Figure 1D) concentrations (p<0.05) were observed in the LPS group compared with the CON group. Supplementing the diet with IA led to a significant rise in the levels of IgA (Figure 1A), IgM, and C3 in the serum in LPS-challenged broilers (p<0.05). Additionally, broilers in the LPS+IA group exhibited notable enhancements in serum IgA and IgM levels (p< 0.05), and displayed a tendency to raise serum IgG and C3 concentrations (p<0.10) in comparison to broilers in the LPS group. The serum complement C4 (Figure 1E) concentration did not show any notable variation among the three groups (p>0.05).

### Hepatic histopathology

**Figure 4:**
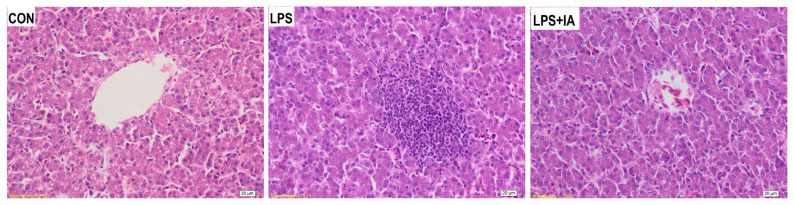
Effects of dietary *Macleaya cordata* isoquinoline alkaloids (IA) on hepatic histopathology in broilers challenged with lipopolysaccharide (LPS). CON, broilers given a basal diet; LPS, LPS-challenged broilers given a basal diet; LPS+IA, LPS-challenged broilers given a basal diet supplemented with 0.6 mg/kg IA extracted from *Macleaya cordata*.

The CON group showed a uniform arrangement of hepatocytes, normal hepatic sinusoid development, and unobvious inflammatory cell exudation though a slight vacuolar steatosis was observed (Figure 4). In the LPS group, a large amount of cell degeneration and inflammatory cell infiltration, fewer lymphocytes and a small number of macrophages were observed in the livers. In the LPS+IA group, hepatocytes were arranged evenly and orderly with normal hepatic sinusoids, while there was a trend of cell division repair and slight exudation of inflammatory cells.

### Hepatic inflammatory factors and caspases activities

**Figure 5:**
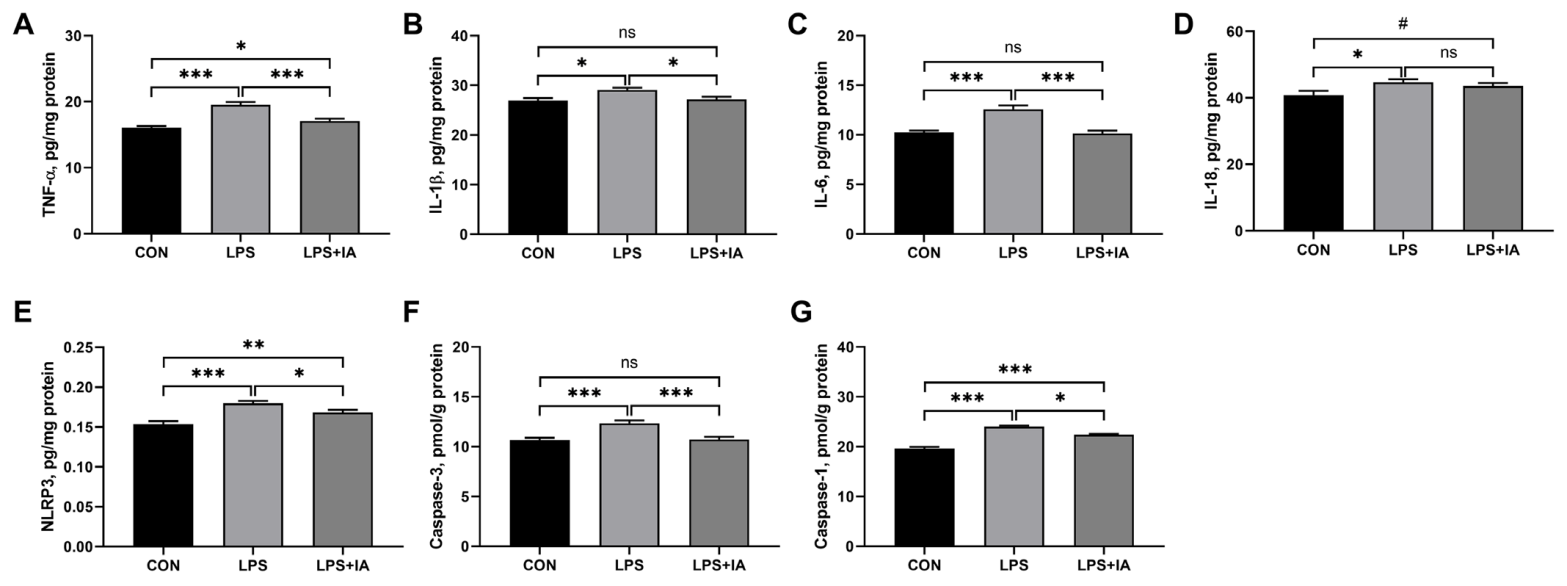
Effects of dietary *Macleaya cordata* isoquinoline alkaloids (IA) on hepatic inflammatory factors and caspases activities in broilers challenged with lipopolysaccharide (LPS). (A) Tumor necrosis factor α (TNF-α); (B) Interleukin-1β (IL-1β); (C) Interleukin-6 (IL-6); (D) Interleukin-18 (IL-18); (E) NOD-like receptor family pyrin domain containing 3 (NLRP3); (F) Caspase-3; (G) Caspase-1. CON, broilers given a basal diet; LPS, LPS-challenged broilers given a basal diet; LPS+IA, LPS-challenged broilers given a basal diet supplemented with 0.6 mg/kg IA extracted from *Macleaya cordata*. All data were expressed as the mean±standard error in the figures. Significant differences were indicated using * p<0.05, ** p<0.01, and *** p<0.001, and ^#^ 0.05<p<0.10 is considered as a significant trend. The “ns” is considered as non-significant differences.

Compared to the CON group, LPS injection resulted in significant increases in hepatic levels of TNF-α (Figure 1A), IL-1β (Figure 1B), IL-6 (Figure 1C), IL-18 (Figure 1D), NLRP3 (Figure 1E), caspase-3 (Figure 1F), and caspase-1 (Figure 1G) (p<0.05) (Figure 5). In LPS-challenged broilers, adding IA to the diet significantly decreased TNF-α, IL-6, IL-1β, NLRP3, caspase-3, and caspase-1 levels in the liver (p<0.05). Moreover, supplementing IA to the diet alleviated LPS-induced increases in liver concentrations of IL-1β, IL-6, and caspase-3 to levels observed in the CON broilers (p>0.05). However, hepatic TNF-α, NLRP3, and caspase-1 levels were significantly higher in LPS+IA group than in CON group (p<0.05), and hepatic IL-18 concentration tended to be higher in LPS+IA group than in CON group (p<0.10).

### Hepatic genes expressions

**Figure 6:**
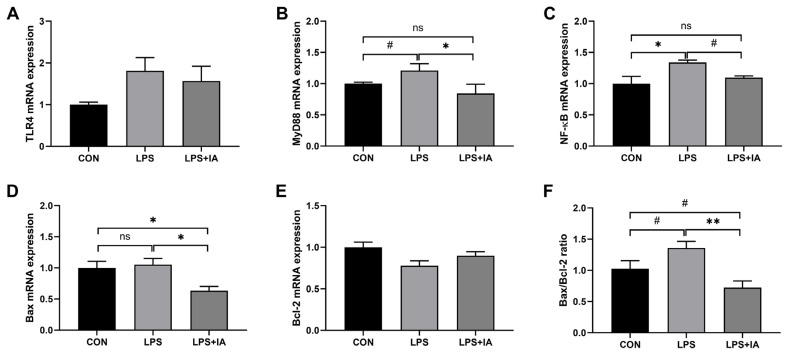
Effects of dietary *Macleaya cordata* isoquinoline alkaloids (IA) on hepatic genes expressions in broilers challenged with lipopolysaccharide (LPS). (A) Toll-like receptor 4 (TLR4); (B) Myeloid differentiation primary response 88 (MyD88); (C) Nuclear factor-kappa B (NF-κB); (D) Bcl-2-associated X (Bax); (E) B-cell-lymphoma-2 (Bcl-2); (F) Bax/Bcl-2 ratio. CON, broilers given a basal diet; LPS, LPS-challenged broilers given a basal diet; LPS+IA, LPS-challenged broilers given a basal diet supplemented with 0.6 mg/kg IA extracted from *Macleaya cordata*. All data were expressed as the mean±standard error in the figures. Significant differences were indicated using * p<0.05 and ** p<0.01, and ^#^ 0.05<p<0.10 is considered as a significant trend. The “ns” is considered as non-significant differences.

As shown in Figure 6, LPS administration significantly up-regulated the mRNA expression of *NF-κB* (Figure 1C) (p< 0.05), and tended to increase the mRNA expressions of *MyD88* (Figure 1B) and *Bax/Bcl-2* ratio (Figure 1F) (p<0.10) in the liver compared with the CON group. In contrast, dietary IA addition significantly decreased the genes expressions of *MyD88*, *Bax* (Figure 1D), and *Bax/Bcl-2* ratio (p<0.05), and tended to decrease *NF-κB* expression in the liver against LPS challenge (p<0.10). Down-regulated mRNA expressions of *Bax* (p<0.05) and *Bax/Bcl-2* ratio (p<0.10) were observed in LPS+IA group compared to CON group, and there were no differences in hepatic *MyD88* and *NF-κB* mRNA expressions between CON and LPS+IA groups (p>0.05). No significant differences were observed in *TLR4* (Figure 1A) and *Bcl-2* (Figure 1E) expressions among the three groups (p>0.05).

## DISCUSSION

In the present study, LPS administration resulted in the increase in serum ALT activity of broilers, which was in accord with previous study [16]. ALT is an enzyme which is abundant in hepatocytes, and its activity is about 3,000 times greater in liver than in serum [22]. Once hepatocytes were disrupted and permeability of the cell membranes increased, it will be released into the bloodstream, causing a great elevation in serum ALT activity [14]. In view of this, serum ALT activity is often used as an important indicator and clinical detection tool to evaluate body hepatocyte injury [23]. Besides, complement C3 was observed to be decreased by LPS injection in current study. Complement C3 is a crucial component of the innate immune system, forming a major host mechanism for potential pathogens clearance in association with other complements [24]. Complement C3 deficiency often occurred together with the diminished regenerative capacity of liver [25], and inflammatory damage to the liver could lead to a reduction in complement C3 concentration [26]. The entrance of LPS to the bloodstream leads to the activation of immune cells, and thereby results in the activation of the complement systems [27], during which the complement C3 will be cleaved to C3a and C3b by C3-convertase [28]. Moreover, complement activation products could induce liver injury via acting on PI3K signaling pathway [29]. Previous study in rats indicated that salvianolic acid A could attenuate liver damage through blocking LPS-induced complement terminal activation [30]. In the present study, LPS broilers also showed obvious pathological alterations, such as cell degeneration and inflammatory cell infiltration, and increased pro-inflammatory cytokines. The results above in this study indicated the successful establishment of the animal model for liver injury induced by intraperitoneal injection of 1 mg/kg BW LPS, which was in line with previous studies [17,18]. On the other hand, dietary supplementation with 0.6 mg/kg IA effectively inhibited LPS injection-induced increases in serum ALT concentration and reduced exudation of inflammatory cells in liver, suggesting that *Macleaya cordata* IA addition could ameliorate the LPS-induced liver injury in broilers.

The liver is a very important, frontline immune tissue, and liver injury can severely impair its functionality [30]. The immunoglobulins and complement components produced by the liver and circulating in the plasma play an important role in defending against bacterial infections and confers passive immunity [31]. Relative to the CON group, LPS stimulation decreased IgM and complement C3 concentrations in the serum, but, interestingly, increased serum IgG concentration in this study. Acting as the initial line of defense against infections, the IgM, which is the first isotype of antibodies to emerge during immune responses, plays a crucial role in host protection [32]. However, the IgG is not only of anti-inflammatory activity, but also can induce pro-inflammatory responses during infection with pathogenic microorganisms [33]. In current study, *Macleaya cordata* IA addition increased IgM and C3 concentrations as well as IgA concentration in serum in LPS-challenged broilers. Immunoglobulin A is the most abundant immunoglobulin synthesized in the body, and is active against several pathogens [34]. Liu et al [7] also observed increased serum IgA, IgM, and C3 concentrations by *Macleaya cordata* IA supplementation in broilers. Therefore, the findings of this study indicated that dietary *Macleaya cordata* IA addition could enhance host defense and immunoregulatory function, and alleviate LPS-induced compromised immune function in broilers.

Inflammatory response is an important precipitating factor in liver damage in modern intensive feeding [35]. In the current study, we also noticed that LPS administration led to an increase in serum LDL and TCHO concentrations, which were regarded two important indictors of cholesterol metabolism. The LDL is responsible for transporting cholesterol from the liver to peripheral tissues, which raises the chances of hyperuricemia and atherosclerosis [36]. When LDL accumulates in the intima, it activates the endothelium to express leukocyte adhesion molecules and chemokines, leading to signaling pathways activation and inflammatory cytokines release [37]. The inflammatory cytokines are essential for the host-response and resistance to pathogens, and their excess secretion also exacerbate necrosis or apoptosis during chronic disease and acute tissue injury [38,39]. Consistently, stimulation of LPS resulted in the increases in pro-inflammatory cytokines (TNF-α, IL-1β, and IL-6) and led to decreased anti-inflammatory cytokine (IL-4) in serum in this study. Besides, LPS challenge increased the secrete of TNF-α, IL-1β, IL-6, and IL-18 in the liver. Tumor necrosis factor-α is produced by macrophages/monocytes during acute inflammation and is responsible for a diverse range of signaling events within cells, thus bring about immune damage of hepatic cells [40]. Interleukin-1 beta and IL-18 are both members of IL-1 superfamily of cytokines, and usually induced by inflammatory signals in a variety of immune cell types [38,41]. In addition, TNF-α and IL-1β can also induce the secretion of IL-6, mediating the impairment of liver cell function and aggravating liver tissue damage [42,43]. However, dietary supplementation with IA from *Macleaya cordata* inhibited the increases in serum and liver TNF-α, IL-1β, and IL-6 concentrations induced by LPS injection to a certain extent. Recent study in broilers also indicated that *Macleaya cordata* IA could reduce IL-1β and IL-6 levels in the liver [7]. Interestingly, we also found that LPS administration significantly increased serum concentration of IL-10, a prototypical anti-inflammatory cytokine produced by CD4 (+) cells and playing a significant part in reducing inflammatory response via inhibiting T cell functions and the upstream activities of antigen presenting cells [44]. Treffkorn et al [45] demonstrated that LPS administration could result in a release of TNF-α, IL-6, and IL-10 in rat liver macrophages. Moreover, previous study also found that increased serum concentration of IL-6 frequently accompanied an increased level of IL-10 in serum under inflammatory conditions [46]. In this study, dietary IA supplementation suppressed the LPS-induced release of IL-10 to the level observed in broilers fed the CON diet. Hence, our findings suggested that 0.6 mg/kg *Macleaya cordata* IA supplementation could relieve LPS challenge-induced liver damage through suppressing systemic and liver inflammation in broilers.

In order to explore the possible mechanisms of *Macleaya cordata* IA relieving LPS-induced hepatic inflammation further, the expressions of the TLR4/MyD88/NF-κB signaling pathway were quantitatively analyzed. The receptor system of TLR4 can recognize LPS molecules, and then initiates the generation of downstream proteins MyD88 to activate the NF-κB signaling pathway, eventually resulting in the production of inflammatory cytokines (including TNF-α, IL-1β, and IL-6) and the induction of cell apoptosis [47]. Previous study has proven that LPS mediated broiler immunopathological alterations of liver through TLR4 signaling pathway [16]. Our present study also found that supplementing *Macleaya cordata* IA to the diet alleviated LPS-induced increases in hepatic MyD88 and NF-κB mRNA expressions, manifesting that Macleaya cordata IA might suppress LPS-induced hepatic inflammatory response through inhibiting TLR4/MyD88/NF-κB signaling pathway. Moreover, the sensitization of the NF-κB signaling pathway is further involved in the expression of the NLRP3 inflammasome [48]. The NLRP3 inflammasome is a multiprotein complex that plays a pivotal role in regulating the innate immune system and inflammatory signaling [47]. Its activation will trigger capase-1-mediated cleavage of IL-1β and IL-18 precursors and cause inflammatory cell death called pyroptosis [49,50]. Consistently, supplementing *Macleaya cordata* IA to the diet alleviated LPS-induced increases in hepatic NLRP3 concentration and caspase-1 activity in the present study. On the other hand, *Macleaya cordata* IA addition down-regulated the gene expressions of Bax and Bax/Bcl-2 ratio in this study. Generally, the pro-apoptotic protein expressed by Bax gene destroys the outer membrane integrity of mitochondria, thus releasing the internal cytochrome C to the cytosol to activate caspases [51]. By contrast, the anti-apoptotic protein Bcl-2, presented in the outer mitochondrial membrane, can combine with Bax protein to form a dimer to neutralize the pro-apoptotic effect of Bax [52]. The increased Bax/Bcl-2 ratio will up-regulate cleavage of caspase-3 and caspase-1, effector caspases initiating the process of cell apoptosis and pyroptosis, respectively [53,54]. Likewise, supplementing IA to the diet alleviated LPS-induced increases in hepatic caspase-3 activity to level observed in the CON broilers. The aforementioned findings indicated that *Macleaya cordata* IA has the ability to inhibit hepatic inflammatory injury caused by LPS through inactivating TLR4/MyD88/NF-κB signaling pathway-mediated cell apoptosis and pyroptosis.

## CONCLUSION

To sum up, dietary addition of 0.6 mg/kg *Macleaya cordata* IA could alleviate LPS-induced liver injury through enhancing immune function and suppressing hepatic inflammatory response via TLR4/MyD88/NF-κB signaling pathway.

## Figures and Tables

**Table 1:** Ingredients composition and nutrient levels of basal diets (as-fed basis)

Items	Content
Ingredients (%)	
Corn	55.91
Soybean meal, 44% CP	13.78
Wheat bran	11.98
Corn starch residue	7.99
Corn gluten meal	3.99
Extruded soybean	1.50
Limestone	1.70
Calcium monophosphate	1.10
L-Lysine HCl	1.00
DL-Methionine	0.20
L-Threonine	0.10
Sodium chloride	0.40
Choline	0.10
Phytase	0.10
Complex enzyme	0.02
Trace mineral premix^1)^	0.10
Vitamin premix^2)^	0.02
Antioxidant	0.02
Total	100
Calculated analysis (%)	
Metabolizable energy (MJ/kg)	12.33
Crude protein	19.47
Crude fat	3.45
Calcium	0.94
Available phosphorus	0.35
Lysine	1.15
Methionine	0.50

1) Provided per kilogram of complete basal diet: 100 mg of Fe as FeSO_4_, 10 mg of Cu as CuSO_4_, 65 mg of Zn as ZnSO_4_, 1.1 mg of I as Ca(IO_3_)_2_, 100 mg of Mn as MnSO_4_ and 0.3 mg of Se as Na_2_SeO_3_.

2) Provided per kilogram of complete basal diet: vitamin D_3_ 3,000 IU, vitamin A 10,000 IU, vitamin K_3_ 1.3 mg, vitamin E 30 IU, biotin 0.2 mg, folic acid 1 mg, niacin 40 mg, D-calcium pantothenate 10 mg, vitamin B_1_ 2.2 mg, vitamin B_2_ 8 mg, vitamin B_3_ 8 mg, vitamin B_6_ 4 mg, and vitamin B_12_ 0.025 mg.

**Table 2:** Primer sequences used for quantitative real-time polymerase chain reaction

Genes	Gene bank No.	Primer sequences^1)^ (5′-3′)
*β-actin*	NM_205518.1	F: TTGGTTTGTCAAGCAAGCGG R: CCCCCACATACTGGCACTTT
*TLR4*	NM_001030693.1	F: AGGCACCTGAGCTTTTCCTC R: TACCAACGTGAGGTTGAGCC
*MyD88*	XM_046910878.1	F: TGATGCCTTCATCTGCTACTG R: TCCCTCCGACACCTTCTTTCTA
*NF-κB*	NM_001396038.1	F: CAGCCCATCTATGACAACCG R: TCAGCCCAGAAACGAACCTC
*Bax*	XM_422067	F: GGTGACAGGGATCGTCACAG R: TAGGCCAGGAACAGGGTGAAG
*Blc-2*	NM_205339.2	F: GCTGCTTTACTCTTGGGGGT R: CTTCAGCACTATCTCGCGGT

*TLR4*, toll-like receptor 4; *MyD88*, myeloid differentiation primary response 88; *NF-κB*, nuclear factor-kappa B; *Bax*, Bcl-2-associated X; *Blc-2*, B-cell-lymphoma-2.

1) F, forward; R, reverse.
